# Dynamic Changes of Ocular Surface in First-Time Contact Lens Wearers and the Effective Factors of Contact Lens Discomfort

**DOI:** 10.3389/fmed.2022.833962

**Published:** 2022-03-11

**Authors:** Yangyang Xu, Zhiqiang Xu, Xupeng Shu, Qiaoli Liu, Yuzhou Wang, Jiahui Xia, Yong Li, Jia Qu, Liang Hu

**Affiliations:** ^1^Eye Hospital and School of Ophthalmology and Optometry, Wenzhou Medical University, Wenzhou, China; ^2^National Clinical Research Center for Ocular Diseases, Wenzhou, China; ^3^Department of Ophthalmology, Xinhua Hospital Affiliated to Shanghai Jiao Tong University School of Medicine, Shanghai, China

**Keywords:** contact lens (CL), dendritic cells, immune response, contact lens discomfort, ocular surface

## Abstract

**Purpose:**

The purpose of the study is to investigate the dynamic changes in ocular surface indicators in first-time contact lens (CL) wearers and identify the most influential factors in CL discomfort (CLD).

**Methods:**

A total of 26 healthy non-CL wearers (26 eyes) were recruited and fitted monthly with disposable hydrogel CLs. Each participant underwent a full ocular surface evaluation, which include Efron grading, tear film breakup time, Schirmer's I test, corneal dendritic cell (DCs) imaging by *in vivo* confocal microscopy (IVCM), and conjunctival microvasculature evaluation by functional slit-lamp biomicroscopy. CLD was assessed using the Ocular Surface Disease Index questionnaire at baseline, 1 week, 1, 3, and 6 months after wearing it and another 6 months after discontinuing it.

**Results:**

Clinical signs and CLD were significantly increased in the first week (*p* < 0.05). The microvascular response and DC activation peaked at the 1-month interval (*p* < 0.05). During CL wear, CLD is positively correlated with corneal staining (*B* = 0.238, *p* = 0.002), papillary conjunctivitis (*B* = 0.245, *p* < 0.001), and microvascular blood flow velocity (*B* = 0.353, *p* < 0.001). After discontinuation, only DC activation remained elevated at 6 months, whereas the other signs recovered.

**Conclusions:**

The first week of CL wear was the main period for the appearance of ocular surface clinical signs, and the first month was the main period for the activation of subclinical inflammation. Corneal staining and conjunctival microvascular response are the main factors affecting CLD. Even if the clinical signs recover after discontinuing wear, subclinical inflammation may persist.

## Introduction

In 2016, Efron (1) emphasized that “contact lens wear is intrinsically inflammatory” and reported that inflammation exists on the ocular surface of normal, asymptomatic contact lens (CL) wearers, which is caused by the inherent factor of the lens. Inflammation related to a CL is characterized by five clinical signs (redness, heat, swelling, discomfort, and discontinuing lens wear) and two subclinical mediators (upregulation of chemical agents and mobilization of cellular elements) (2).

Some studies have reported dynamic changes in ocular surface inflammation in CL wearers. Alzahrani et al. (3) and Liu et al. (4) found that the density of corneal dendritic cells (DCs) increased significantly from 1 week to 1 month after CL wear and decreased gradually after 1 month. Sorbara et al. (5) reported that after 2 weeks of lens wear, the flow velocity of the conjunctival blood vessels increased in both silicone hydrogel and hydrogel lens wearers. Maldonado-Codina et al. (6) observed that corneal limbal hyperemia increased significantly after 2 weeks of lens wear, but decreased after the fourth week. These studies have described dynamic inflammatory changes with time at only one level, but the overall longitudinal observation of ocular surface clinical signs and subclinical mediators simultaneously has not yet been reported. In addition, changes in ocular surface inflammatory indicators after CL wear and after stopping wear in first-time CL wearers have not yet been reported. Finally, the dynamic changes of discomfort, and also the relationship between discomfort and ocular surface inflammation, also need further study for investigation, which was suggested by Efron (2).

The International Workshop on Contact Lens Discomfort convened by the Tear Film and Ocular Surface (TFOS) Society proposed that inflammation may play a role in the etiology of discomfort experienced during uncomplicated CL wear (7). CL discomfort (CLD) is the most common event reported by CL wearers to ophthalmologists and optometrists. Reportedly, 50–75% of CL wearers experience CLD (8, 9). In addition, 12–51% of CL wearers discontinue or drop-off wearing CLs due to CLD (10, 11). Moreover, according to Richdale, CLD is the major issue for CL dropout (12). Steffen and Barr (13) suggested that tinted lenses increase ocular discomfort compared to that associated with conventional clear lenses. Hydrogels remain the major materials for tinted lenses. The study focused on CLD in hydrogel CL wearers and was clinically significant. Currently, the etiology and clinical signs of CLD are not fully understood, which makes it difficult for ophthalmologists and optometrists to manage it.

As described previously, several studies have observed ocular surface responses to uncomplicated CL wear, such as conjunctival or limbal redness, DC activation, and microvascular blood flow response. Whereas, these studies provided valuable insight into the reaction of the eye to CL wear and the link to CLD, there has not been a comprehensive model that combines all of these elements to serve as a basis for investigating the relationship between these various measures of inflammation and discomfort during uncomplicated CL wear.

This study aimed to evaluate ocular surface inflammatory indicators (corneal DC, conjunctival blood vessel flow, and clinical signs) and CLD in first-time CL wearers to reveal the dynamic changes in the ocular surface and the most influential factors of ocular surface clinical and subclinical inflammation in CLD. With this, we hope that this study will provide a possible reference for CLD clinical management in first-time CL wearers.

## Methods

### Study Design and Participants

This was a longitudinal interventional cohort study, approved by the Human Sciences Ethical Committee of the Eye Hospital at Wenzhou Medical University (approval number: KYK2018-23) and followed the tenets of the Declaration of Helsinki (trial registration: ChiCTR, ChiCTR1800018001), registered on August 26, 2018, https://www.chictr.org.cn/edit.aspx?pid=29659&htm=4). This study was conducted from September 2018 to December 2019 at Wenzhou Medical University Eye Hospital. Written informed consent was obtained from all participants after the complete explanation of the study's content.

We used G^*^Power (version 3.1, Düsseldorf University, Germany) (14) to calculate the sample size, and the maximum sample size was used as a reference. Based on the differences in conjunctival vascular blood flow velocity changes within five visits, 20 participants were needed to determine the differences with a detection power of 0.8 and an effective size of 0.25.

**Figure 1:**
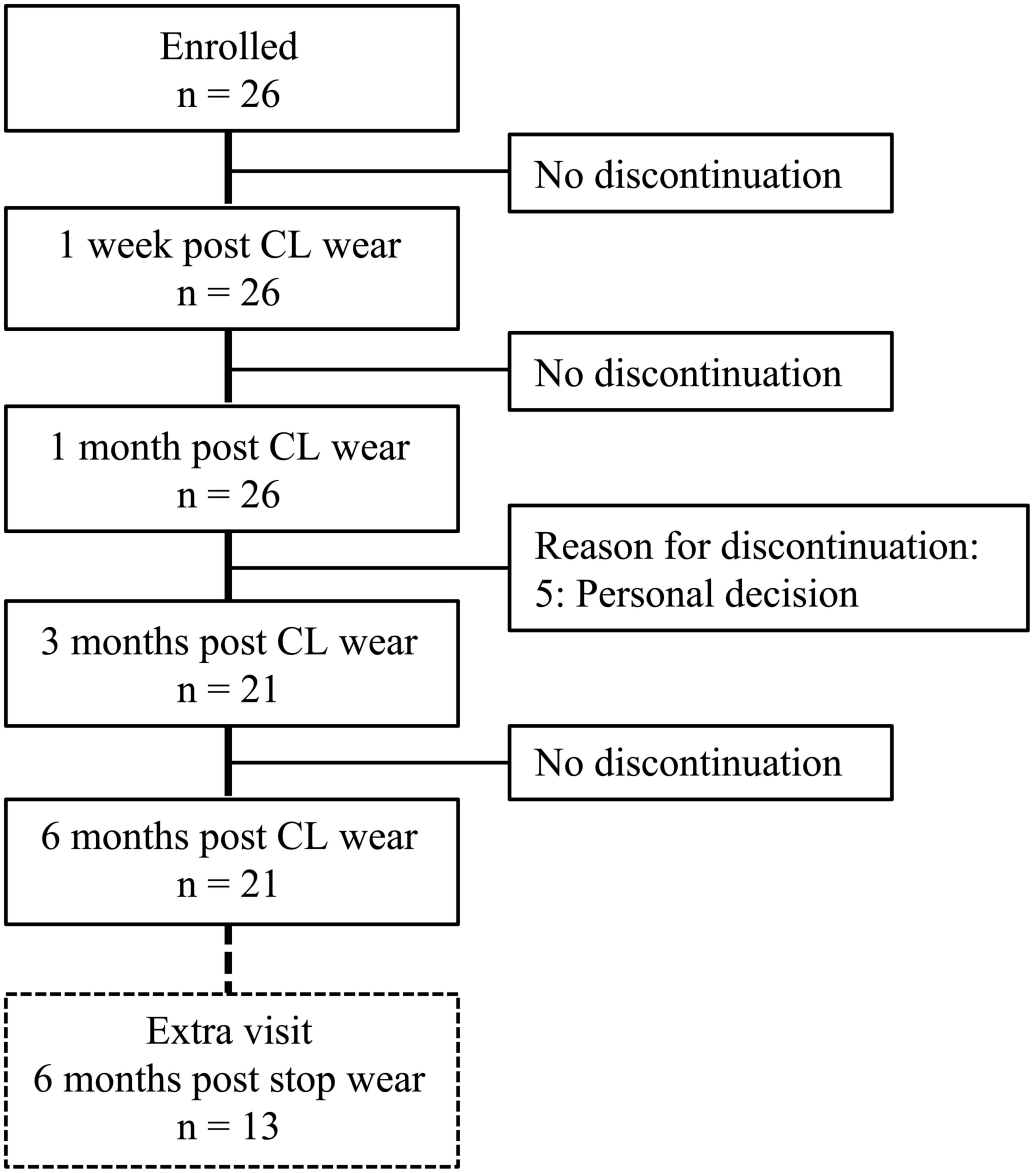
Flow diagram of the study. OSDI, ocular surface disease index; CL, contact lens.

The selected participants who had never worn CLs and satisfied the inclusion criteria (Table 1) were included. All enrolled participants underwent lens fitting by a CL specialist with 5 years of working experience. The participants were instructed not to wear the lens until they achieved a good lens fit. Monthly disposable CLs (SofLens 59^®^, BAUSCH & LOMB Inc., NY, USA; Supplementary Table S1) for daily wear and ReNu^®^ Multiplus Solution (BAUSCH & LOMB^®^ Inc., NY, USA) were provided. The CL wear modality was an 8-h daily wear, 5 days per week, and replaced every 1 month (the lens was removed each evening, cleaned, and then stored in ReNu^®^ Multiplus Solution). Online WeChat groups and paper recording forms were used to supervise and self-report the participants' daily routines to ensure that each participant followed the CL wear modality. All participants wore CLs in both eyes for 6 months and then discontinued wearing them. This study consisted of five basic visits (baseline, 1 week, 1, 3, and 6 months) and one extra visit (6 months after discontinuing wear) if the participants were suitable and available (those who were still wearing CLs after 6 months would be unsuitable for the extra visit). A total of twenty-six participants were enrolled, 21 completed 3 and 6 months of follow-up, and 13 participants were available for extra follow-up after discontinuing CL wear (Figure 1).

**Table 1:** Inclusion and exclusion criteria.

**Inclusion criteria**	**Exclusion criteria**
Age 18–30 years	Systemic or ocular allergies which might affect CL wear
Healthy participants	Systemic or ocular disease which might affect CL wear
No history of CL wear	Systemic or ocular medication use which might affect CL wear
Sphere power: plano to−10.00 D	History of ocular surface surgery (refractive surgery, etc.)
Cylinder power: plano to−1.50 D	Active ocular infection
Corrected VA better than 20/25^#^	Dry eye^*^ (OSDI ≥ 13 + tear film breakup time (TBUT) <5 s)
Willingness to wear soft CL	Significant ocular surface signs (Efron grade > 2.0) and need clinical intervention
Able to participate in the study	Smoker or/and alcoholism
Signature in the informed consent form	Pregnancy or lactation

*VA, visual acuity; TBUT, tear film breakup time; OSDI, ocular surface disease index*.

# *Corrected by CL, VA better than 20 out of 25 and without any self-reported complaint of vision-related discomfort*.

* *Dry eye definition and diagnosis according to “new perspectives on dry eye definition and diagnosis: a consensus report by the Asia Dry Eye Society” (15). Dry eye diagnosis must follow two conditions: OSDI ≥ 13 and TBUT <5 s*.

### Ocular Surface Clinical Signs

Ocular surface signs were graded according to the Efron grading scale by three masked ophthalmologists with 5 years of working experience (Y. X., Z. X., and X. S.) from six images of the anterior segment, which were taken *via* a functional slit-lamp biomicroscope (FSLB) by a blinded researcher (J. X.). These images included the temporal bulbar conjunctiva, cornea, lower eyelid margin, superior palpebral conjunctiva, corneal staining, and bulbar conjunctiva staining. Eight indices in the Efron grading scale were used, which include conjunctival redness, limbal redness, corneal neovascularization, meibomian gland dysfunction (MGD), blepharitis, corneal staining, conjunctival staining, and papillary conjunctivitis (each index grade ranged from 0 to 4 with 0.1-unit step) (16). The mean value of each sign grade for each participant by these three ophthalmologists was used for the statistical analyses.

Tear film breakup time and Schirmer's I tests (fluorescein strips and Schirmer's strips: Tianjin Jingming New Technological Development Co. Ltd, Tianjin, China) were conducted without anesthesia and were measured in a standardized manner by a blinded ophthalmologist (Y. X.).

### Ocular Surface Microvascular Response

Conjunctival microvascular evaluation was performed using the FSLB, which consisted of a slit-lamp biomicroscope (SLM-4ER; Kanghua, Inc., Chongqing, China) equipped with a Canon 60D digital camera (Canon Inc., Melville, NY, USA) (maximum magnification 175 × , field of view 1.131 mm × 0.849 mm, image size 640 × 480 pixels, resolution 3.91 μm, and frame rate 60 Hz). The protocol and parameter calculations used in this study have been previously described by Chen et al. (17). Initially, the FSLB imaged six fields of the temporal bulbar conjunctiva located ~1 mm radially from the limbus (18). The FSLB system recorded the blood flow of the conjunctival microvasculature *via* high-speed video capture and compensated for the rotation of the eyes through a video image processing program (MATLAB, ImageJ, and Benoit). The microvasculature parameters that include axial blood flow velocity (Va, mm/s) and vessel density (Dbox) were then calculated. The images and videos were captured by a blinded researcher (J. X.) and analyzed by another researcher (X. S.). The mean values of the six fields were then used for the statistical analyses.

### Ocular Surface Immune Cells

**Figure 2:**
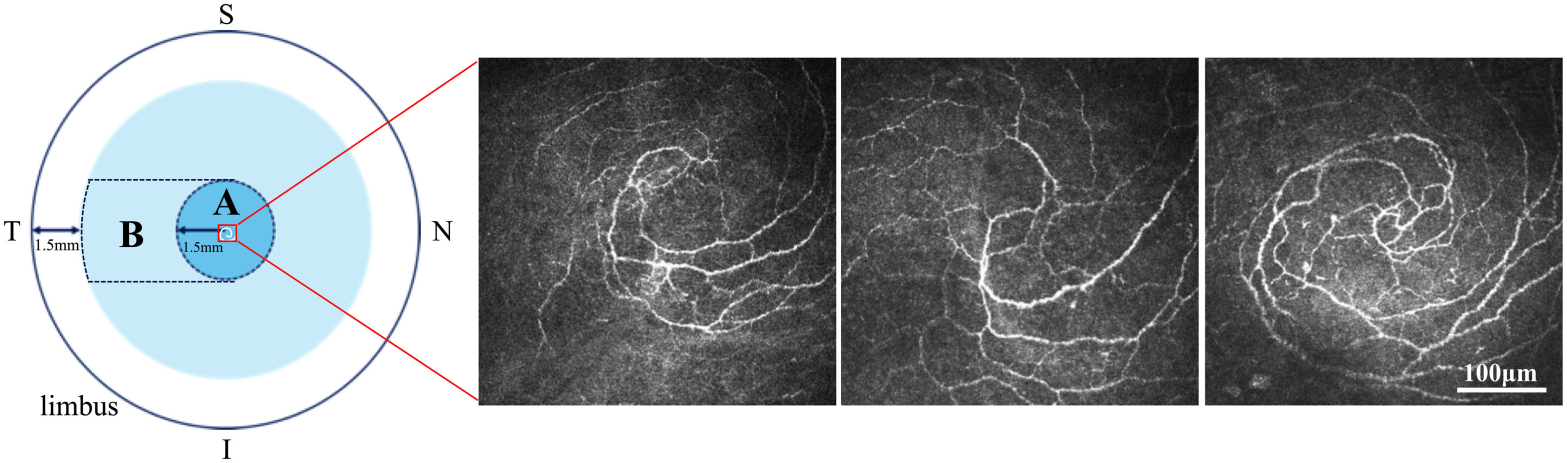
Definition of central and peripheral cornea. The central cornea (A) was defined as the 1.5-mm area around the nerve spiral structure. The peripheral cornea (B) was defined as the area from the temporal side of the central cornea (A) to 1.5 mm away from the limbus. The three IVCM images showed the nerve spiral structure in three different participants of this study. S, Superior; I, Inferior; N, Nasal; T, Temporal. *Scale bar:* 100 μm.

Corneal DCs were imaged using *in vivo* confocal microscopy (IVCM) (Heidelberg Retina Tomography II with a Rostock Cornea Module: Heidelberg Engineering Corp., Dossenheim, Germany), which comprised 384 × 384 pixels with a 400 μm × 400 μm coverage area (19). Both central and peripheral corneas were imaged, with central and peripheral cornea definitions, as described in Figure 2. The central cornea was defined as an area of 1.5 mm around the spiral nerve structure. The peripheral cornea was defined as the area from the temporal side of the central cornea, 1.5 mm away from the limbus. To verify corneal integrity, a slit-lamp examination was performed pre- and post-IVCM. IVCM was focused at a corneal depth of 50–80 mm throughout the examination. A masked ophthalmologist with a 3-year working experience (Q. L.) took ~150 digital images from posterior to anterior through the cornea in the central and peripheral areas separately, totaling ~300 images. Then, the same doctor selected three high-quality and non-overlapping images with the maximum number of target cell selected by the same doctor. The specific practice steps and parameter calculations were described in a previous study by Hu et al. (20). DC density (cells/mm^2^) was calculated using the manual model in ImageJ software (Heidelberg Engineering GmbH Version 1.51w, NIH, MD, USA). The 10 best representative cell images were selected from three preceding high-quality images of the central and peripheral cornea separately. Cell morphological parameters that include DC area (μm^2^) and number of dendrites per DC were also calculated using the ImageJ software. DC density and cell morphology analyses were conducted by two blinded researchers (Y. W. and Y. L.). The mean value of the results of these two researchers was recorded as the final result.

### CLD

The Ocular Surface Disease Index (OSDI) questionnaire [a validated Chinese version of the OSDI (21)] was used to analyze CLD, as the OSDI is one of the most widely used, stable, and reliable surveys in research concerning CL-related comfort (22). The score ranged from 0 to 100 with 1-unit steps.

### Examination Procedures

The procedures were performed on the right eye only, from the least to the most invasive (1. OSDI questionnaire, 2. FSLB conjunctival vascular evaluation, and 3. FSLB anterior segment images capture—for Efron grading, 4. TBUT, 5. Schirmer's I test, 6. IVCM corneal DC images capture) and within 4 h after awakening or 3 h after CL wear. All visits were performed between 10:00 a.m. and 1:30 p.m. To eliminate the impact of lens removal on the ocular surface microenvironment, except for Schirmer's I test and IVCM, other examinations were conducted with CL wear. The temperature of the examination room was maintained at 20–21°C and 50–60% humidity. At each follow-up visit, the examination sequence and contents were the same.

### Statistical Analysis

Data were analyzed using SPSS version 23 (IBM Corp., NY, USA) and GraphPad Prism version 7 (GraphPad Software Inc., NC, USA). Data normality was assessed by the combination of the Shapiro–Wilk test and histogram. Values are described as the mean ± standard deviation or median (interquartile range), which depends on normality. For the five basic visits, one-way repeated measures analysis of variance (ANOVA) was used to test the variation of parameters with time, whereas the Sidak method was used for pairwise comparison between each time point. The variables were standardized to remove unit dimensions before the following analysis. The effective factors for CLD (OSDI score) over time were analyzed using a generalized estimating equation (GEE). Then, partial correlation was used to test the relationships between effective factors and other variables, with participant demographics and time points as the controlled variables. For one extra visit, paired Student's *t*-test (in case of normal distribution) or Wilcoxon signed-rank test (for paired comparison of non-normally distributed data) was used to determine the difference between the baseline and extra visits and 6 months vs. extra visits. The hypothesis tests were two-sided, and *p* < 0.05 indicated statistical significance.

## Results

### Participants' Demographics

There were 26 participants (26 eyes) at the baseline, with nine (34.6%) men and 17 (65.4%) women. Supplementary Table S2 presents the demographic characteristics of the participants. A total of 21 participants (21 eyes, 81%) completed the five-time basic follow-up and 13 (13 eyes, 50%) completed an additional follow-up.

### The Changes in Clinical Signs, Microvascular Response, Immune Cells, and CLD From Baseline to 6 Months Post-CL Wear

**Figure 3:**
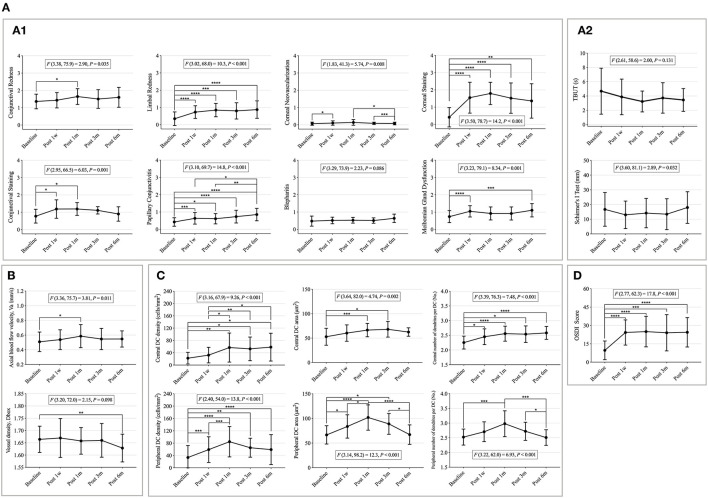
The changes of clinical signs, microvascular response, immune cells, and contact lens discomfort (CLD) from the baseline to 6 months post-CL wear. **(A)** Changes of clinical signs with time. **(A1)** Changes of eight Efron grading indices with time. **(A2)** Changes of tear film related indexes with time. **(B)** Changes of conjunctival vascular indexes with time. **(C)** Changes of corneal dendritic cell (DC)-related indices with time. **(D)** Changes of CLD with time. Points and error bars indicated mean ± standard deviation (SD). DC, dendritic cells; OSDI, ocular surface disease index; *F*(DFn, DFd) and *P*, one-way repeated measures ANOVA; DFn, degrees of freedom numerator; DFd, degrees of freedom denominator. *p < 0.05; **p < 0.01; ***p < 0.005; ****p < 0.001, *P*, Sidak method was used for pairwise comparison between each time point.

Seven Efron grading indices (conjunctival and limbal redness, corneal neovascularization, corneal and conjunctival staining, papillary conjunctivitis, and MGD) and Schirmer's I test significantly changed with time (*p* < 0.05) (Figures 3A1,A2). Limbal redness, corneal neovascularization, corneal and conjunctival staining, papillary conjunctivitis, and MGD scores increased significantly 1 week after CL wear compared to that at the baseline (*p* < 0.05); corneal and conjunctival staining showed a decreasing trend from the third month but without statistical significance (Figure 3A1). Schirmer's I test showed a tendency to decrease first (1 week) and increase later (6 months) (Figure 3A2).

Meanwhile, the Va value significantly increased after 1 month of CL wear (baseline vs. 1 month: *p* = 0.037) and then decreased, but without statistical significance (Figure 3B).

Corneal DCs are activated over time, particularly in the peripheral cornea. The peripheral corneal DC density, DC area, and number of dendrites significantly increased 1 week after CL wear and peaked at 1 month, then decreased after 3 months of CL wear (Figure 3C). The central corneal DC indicators continually increased from the baseline after CL wear (*p* < 0.05) (Figure 3C).

The OSDI scores differed over time (*F* = 17.8, *p* < 0.001) (Figure 3D). The OSDI scores increased significantly after 1 week of CL wear (baseline vs. 1 week, *p* < 0.001; baseline vs. 1 month, *p* < 0.001; baseline vs. 3 months, *p* = 0.003; and baseline vs. 6 months, *p* < 0.001). No significant differences were found among the other time points.

The detailed values of the clinical signs, microvascular responses, immune cells, and CLD at each time point are shown in Supplementary Table S3. The sample images are listed in Supplementary Figure S1.

### The Effect of Demographics, Clinical Signs, Microvascular Response, and Immune Cells on CLD During CL Wear

Generalized estimating equation revealed that increased OSDI scores were significantly associated with increased corneal staining (*B* = 0.238, *p* = 0.002), more papillary conjunctivitis (*B* = 0.245, *p* < 0.001), higher Va (*B* = 0.353, *p* < 0.001), and higher CL power (*B* = 0.324, *p* < 0.026) (Supplementary Table S4). Other variables did not significantly affect CLD.

**Figure 4:**
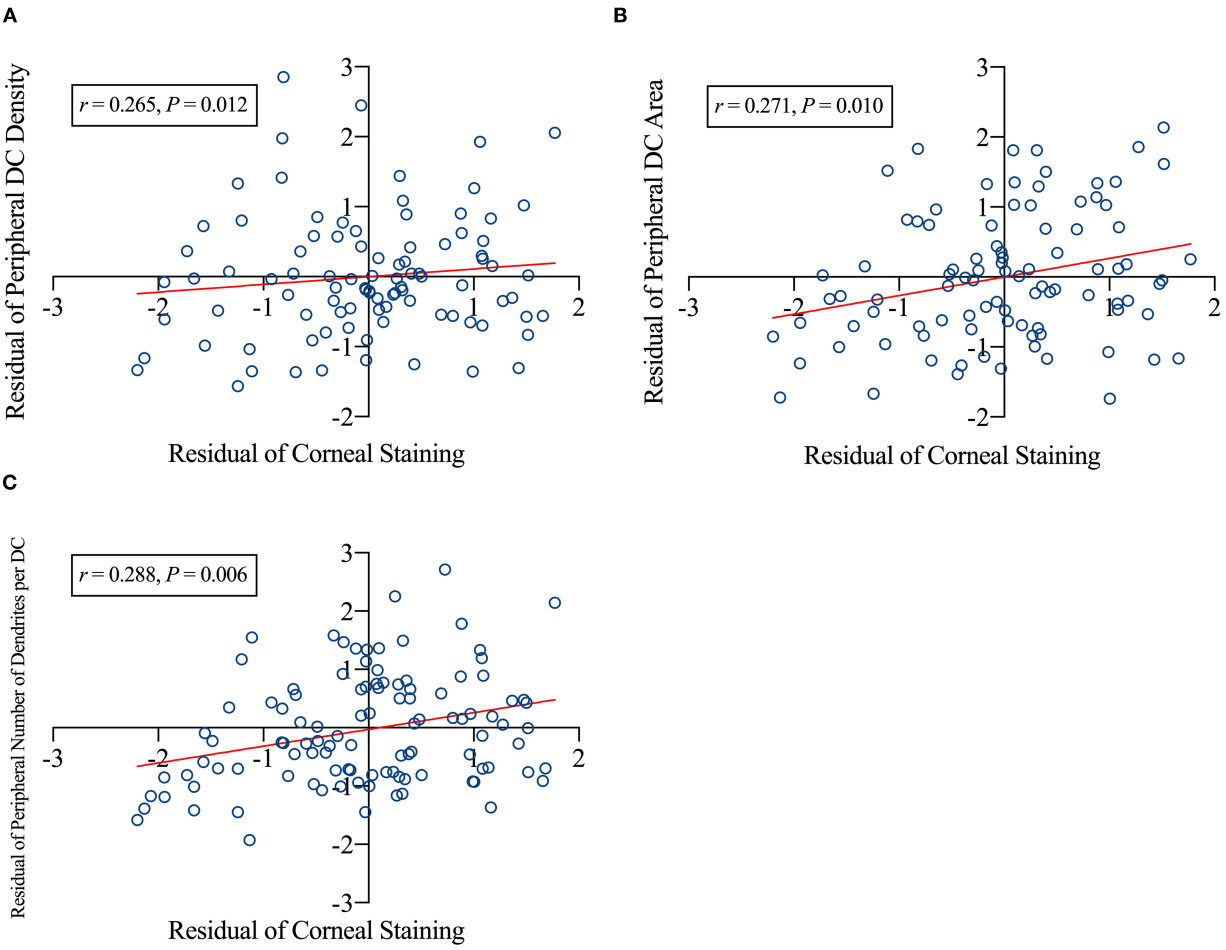
Factors related to corneal staining. The corneal staining scores positively related to peripheral dendritic cell (DC) density **(A)**, peripheral DC area **(B)**, and number of dendrites per DC of the peripheral cornea **(C)**. DC, dendritic cells; *r*, correlation coefficient. *p*-Values indicated the significance of the partial correlation. The CL power, age, sex, and time were the controlled variables. A scatter plot was created using the residuals of the variables obtained *via* regression analysis.

Furthermore, partial correlation revealed that greater corneal staining was significantly associated with higher peripheral DC density (*r* = 0.265, *p* = 0.012; Figure 4A), larger peripheral DC area (*r* = 0.271, *p* = 0.010; Figure 4B), and more dendrites per DC in the peripheral cornea (*r* = 0.288, *p* = 0.006, Figure 4C).

### The Recovery of Clinical Signs, Microvascular Response, Immune Cells, and CLD After 6 Months of Discontinuing CL Wear

The data of 13 participants who participated in the additional follow-up were used for analysis.

**Figure 5:**
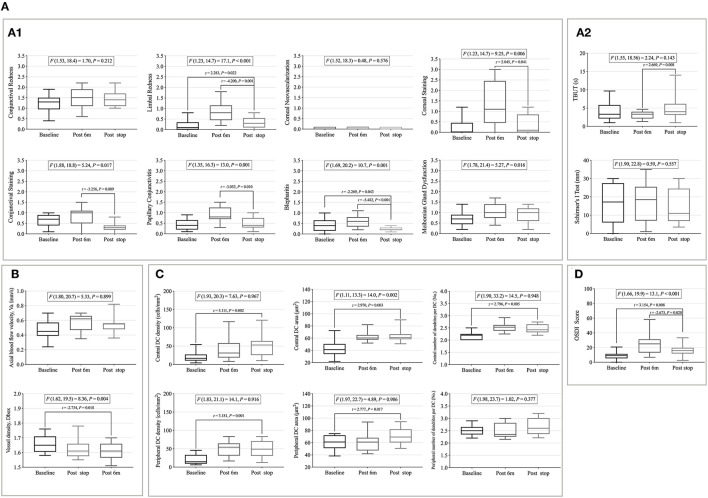
The recovery of clinical signs, microvascular response, immune cells, and contact lens discomfort (CLD) after 6 months after ceasing CL wear. **(A)** The recovery of clinical signs after 6 months after ceasing CL wear. **(A1)** Eight Efron grading indicators. **(A2)** Tear film related indexes. **(B)** The recovery of conjunctival vascular indicators after 6 months. **(C)** The recovery of corneal dendritic cell (DC)-related indicators after 6 months. **(D)** The recovery of CLD after 6 months. Whiskers indicated max and min. DC, dendritic cells; OSDI, ocular surface disease index; *t* and *P*, Paired Student's *t*-test; *z* and *P*, Wilcoxon signed-rank test.

Among the clinical sign indicators, limbal redness (*t* = −4.200, *p* = 0.001), corneal staining (*z* = 2.045, *p* = 0.041), conjunctival staining (*t* = −3.256, *p* = 0.009), papillary conjunctivitis (*t* = −3.052, *p* = 0.010), blepharitis (*t* = −5.482, *p* < 0.001), and TBUT (*z* = 2.669, *p* = 0.008) significantly recovered after discontinuing CL wear compared to the 6-month CL wear (Figures 5A1,A2).

No decrease in the microvascular response and immune cell activation was found after stopping CL wear, and it remained at the level of 6-month CL wear (Figures 5B,C).

The OSDI scores decreased after ceasing wear (*t* = −2.673, *p* = 0.020) but were still higher than the baseline value (*t* = 3.154, *p* = 0.008) (Figure 5D).

## Discussion

Here, we report our longitudinal observations of the ocular surface of 21 first-time CL wearers. To our knowledge, this is the first study to observe the overall changes in inflammation related to ocular surface signs and CLD from first-time CL wear to the cessation of CL wear. Several ocular surface clinical signs, microvascular response, immune cell activation, and CLD significantly increased after CL wear, especially in the first week and first month. We also reported that increased vessel blood flow and corneal staining were the main factors that affect the increase in CLD. Moreover, after discontinuing CL wear, the clinical signs returned to the baseline level, but the activation of immune cells persisted even 6 months after CL wear.

### Changes of Clinical Signs With Time

An increase in ocular surface clinical signs after CL wear has been observed in many previous studies (23–28). Within this study, we observed that during successful CL wear [CL wear without unbearable discomfort and complications requiring clinical treatment and/or intervention, such as severe corneal staining, and papillary conjunctivitis (29, 30)], a progressive increase in ocular surface staining was found in the first week and reached its peak in the first month, but then decreased by the third month. Morgan et al. (23) also observed a similar trend in daily disposable silicone hydrogel (SiH) lens wearers. Best et al. (24) observed a significant increase in staining after 6 months of monthly disposable SiH lens wear in participants wearing new CLs. Ocular surface staining has been attributed to the mechanical effect induced by the lenses (25), to epithelial microtrauma induced by mucin balls, leaving small surface depressions where fluorescein accumulates (26), and to the toxicity of the CL solution (27, 28). Because compromise to the corneal epithelium may place CL wearers at a higher risk for CLD, factors associated with staining should be carefully considered when monitoring CLs, especially in the first month.

In this study, compared with ocular surface staining, the limbal redness and papillary conjunctivitis grades increased progressively after lens wear and decreased significantly to the baseline level after ceasing wear; however, the changes in these two ocular signs were only from normal to slight redness, and the grades were < 1.5, which was not clinically significant. This was consistent with Chao's report that daily disposable CL wear induced lower levels of signs of inflammation (31). In this study, the observation after CL wear was only up to 6 months, whereas the following studies reported long-term (>6 months) changes in the ocular surface. In 18-month follow-up study, Santodomingo-Rubido et al. (32) found that limbal redness increased over the first 6 months, papillary conjunctivitis grades increased over the first 3 months, and both stabilized at that level thereafter. Morgan et al. (23) observed a small increase in papillary conjunctivitis grades in 30 SiH CL wearers in the first month of the 1-year follow-up, with no changes in limbal redness. Papillary conjunctivitis is an inflammatory condition that can be mechanically or immunologically mediated (33). In CL wearers, the etiology appears to be principally mechanical and may be related to changes in surface wetness (34). Therefore, the condition generally resolves very quickly, simply by ceasing CL wear or changing to daily disposable CLs for a period of ~2 weeks (34). This was also observed in the results of this study after stopping the CL wear.

Previous studies have suggested that chronic hypoxic damage caused by CL wear may have long-term effects on ocular surface health (35). Limbal hyperemia may also serve as an early indicator of inflammatory response (36). The limbal response to CL wear results not only in new vessel growth but also because the limbus is the only source of new corneal epithelial cells. Corneal damage (as seen by staining) may activate corneal stem cells in the limbus to stabilize corneal health.

### Changes of Microvascular Response With Time

This was the first long-term longitudinal study to observe the microvascular response of the bulbar conjunctiva after CL wear, which complemented the short-term (6 h) and cross-sectional results (18, 37, 38). The blood flow velocity showed a peak at 1 month after CL wear, which was similar to the change trend of DC of the peripheral cornea. This suggests that a possible synergistic effect of the microvascular response and immune cell activation may exist on the ocular surface. Moreover, the conjunctival microvascular blood flow velocity was around 0.50 mm/s in non-CL participants and 0.57–0.60 mm/s in long-term or habitual CL wearers (37), which were similar to the value in this study (baseline: 0.52 mm/s, after 6 months: 0.57 mm/s). The microvascular response after CL wear is slight and progressive. Previous studies (18, 37, 38) suggested that a ceiling value may exist (< 0.70 mm/s) of blood flow velocity for long-term CL wearers without complications [such as corneal staining, papillary conjunctivitis, CLD (29)]. However, whether the blood flow velocity could be used as a reference for clinical prediction and monitoring of CL-related discomfort requires further study.

### Changes of Immune Cells Over Time

The changes in inflammation-related ocular surface signs after CL wear mentioned above were minimal in this study, but changes at the cell levels were statistically significant after CL wear. As proposed by Chao (31) in the review, the changes in DCs within 1 month and after discontinuation have never been reported, and our study provides reference data for this. Compared with the progressive increase in DC parameters of the central cornea, the DC density and morphological parameters in the peripheral cornea resulted in an obvious peak at 1 month after CL wear. The difference in the changes in DCs between the central and peripheral parts may be related to the distribution characteristics, and Modeen reported that the DC density at the peripheral cornea is 3-folds higher than that at the central cornea (39). Moreover, similar to the results of this study, the vast majority of research on corneal DC densities in CL wearers suggest an increased number of DCs in the central cornea (3, 40, 41) regardless of lens material (hydrogel or SiH lens) or solution (multipurpose or hydrogen peroxide). This increase is presumed to occur because of the migration of DCs into the imaging field. However, an apparent increase in the density of DCs could also occur as a result of a change in the morphology of DCs, which develop dendrites upon maturation once they come into contact with a presentable antigen (42). Similarly, the migration of resident DCs to the central cornea in response to CL wear adaptation has been described in humans (40), which indicates a subclinical inflammatory response. Immature DCs are equipped to capture antigens, whereas mature DCs are able to sensitize native T cells through major histocompatibility complex molecules and secretion of interleukin-12, and also costimulatory molecules, and thus represent an integral part of the immune system (43). In this study, a reduction in DC density and morphology indicators was observed in the third month of CL wear in the peripheral cornea (Figure 3C). Various hypotheses can be proposed to explain the gradual reduction in DC parameters of the peripheral cornea. Since activated DCs migrate to secondary lymphoid tissue to perform antigen presentation (42), the apparent decrease in numbers after 1 month could be explained by the migration of activated cells away from the peripheral cornea on their journey. Future work should investigate whether short-term subclinical findings can predict long-term CL adverse event rates and whether these observations can identify individuals who are at heightened risk of developing CL-related complications [such as corneal staining, papillary conjunctivitis, and CLD (29)].

Most previous longitudinal studies on corneal DCs focused only on the central region of the cornea, and few assessed the peripheral cornea (44), which may be due to the difficulty of accurate localization during IVCM. In the past, real-time feedback imaging from a side-mounted charge-coupled device camera to an IVCM has often been used as the primary reference for localization (3). In this study, the special spiral structure of the corneal nerve [which has been described by Zhivov et al. (43)] was used as the tissue marker of localization. We used the feedback imaging described in a previous study (3) to roughly locate the central section and then slightly moved to the target nerve structure, which was the starting point for image capture. Corneal DCs migrate along the corneal nerves and cornea (45); therefore, they are primarily distributed around the nerves. If we simply used the center of the cornea as the central area for the DC observation, deviations would inevitably occur because the DCs are not evenly distributed in the central cornea. The confluence of nerves in the central inferior cornea was used as the central marker, which was more in line with the distribution characteristics of corneal DCs. This localization method is worth popularizing in the future studies.

### The Effective Factors on CLD

Contact lens comfort is a major determinant of successful lens use. In this study, increased discomfort was associated with increased corneal staining and higher conjunctival blood flow velocity. This finding also indirectly supported the hypothesis suggested by the International Workshop on Contact Lens Discomfort convened by the TFOS Society that inflammation might be one of the etiologies of CLD (7).

Microvascular flow velocity may be a part of subclinical inflammation related to CL wear. Zhang et al. reported that an increase in the blood flow velocity represents inflammation of the ocular surface (46). Chen et al. (17) reported that adapted CL wearers exhibit changes in vascular responses, which may be beneficial. For conjunctival vascular responses in the long-term, experienced CL wearers increased even when they did not wear the CL (38, 47). As the immune system in the bulbar conjunctiva was upregulated, such vascular responses in CL wearers keep the eye prepared against any extrinsic noxious challenge. Efron (1) also suggested that wearing CLs may lead to chronic and low-grade subclinical inflammation, which may have a positive and protective effect on successful CL wearers. In accordance with this, we assume that increased vascular velocity brings more inflammatory factors, which further activate immune cells, thus mobilizing the inflammatory response on the ocular surface; however, this hypothesis requires further study of inflammatory factors to confirm.

As discussed earlier, ocular surface staining (corneal and conjunctival) is mainly contributed by mechanical damage from the CL. Additionally, the significant positive correlation between staining and DC activation (Figure 5) observed in this study suggests that corneal staining by CL wear is likely the main driving factor that activates the corneal immune response. These results also indicate that DC activation is adaptive in CL wearers to compensate for the effect of CL-induced ocular surface damage. CL symptomatology has recently been associated with neuropathic corneal pain (48) using a model involving the interlink between the immune system and ocular discomfort. Recent work also suggests a role for DC activation in the cornea as part of this immune “crosstalk” (49).

### Lens and Participant Selection

In this study, hydrogel CLs were provided to the participants. Steffen and Barr (13) suggested that tinted lenses increase ocular discomfort compared with conventional clear lenses. Hydrogels remain the major materials for tinted lenses. Therefore, this study focused on the former finding that CLD in hydrogel CL wearers is clinically significant. Additionally, adults aged 18–30 years are the main group of newcomers to CL wear with the purpose of improving appearance (50). Comfort of CL wear and the occurrence of complications are the main concerns of this group of people (10). Therefore, the research objective of this study was mainly concentrated in this population, and it is hoped that the results of this study could provide a reference for the clinical management of this population.

### Limitation and Prospect

Efron (2) established a CL-associated ocular surface inflammation model with ocular surface comfort as the central defining element, followed by clinical signs and subclinical mediators. This study adopted a design similar to this model and created a sophisticated and comprehensive experimental paradigm, whereby all of these components were measured contemporaneously and longitudinally in a cohort of first-time CL wearers. The first study, based on this model, has some limitations. First, this study lacked a control group. The participants in this study were instructed to wear CLs in both eyes to improve the feasibility of long-term follow-up and the rationality of research ethics. Further, to eliminate the influence of the correlation between the binocular data of the same participant in the research results, only the data of the right eye of the participants were obtained for the analysis. Moreover, the initial design of this study was a self-controlled longitudinal study, and comparisons were made between individuals. The main purpose of this study was to investigate dynamic changes in ocular surface indicators in first-time CL wearers, and the self-control study design was sufficient to reveal such changes. We would set a control group for further study. Second, only one kind of CL was used in this study. The aim of this study was to determine how the ocular surface changes during long-term lens wear and whether there is a potential relationship with discomfort, using a commonly used hydrogel soft CL. Whether such changes would be affected by different types of lenses is our next question. Finally, the sample size was small but was a result of a long follow-up period, complicated examination procedures that must be performed in one visit, and strict inclusion criteria. A more simplified follow-up design will be implemented in future studies. In the future, sophisticated experimental paradigms (such as FSLB and IVCM) could be translated into the clinical domain so that practitioners can attain more sensitive techniques for measuring the ocular response to CL wear. Such observations would form the basis for devising strategies for dampening the intrinsic inflammatory response of CL use to enhance CL comfort.

## Conclusions

The first week of CL wear was the main period for the appearance of clinical signs on the ocular surface, and the first month was the period of activation of subclinical inflammation. Corneal staining, papillary conjunctivitis, and conjunctival microvascular response were the main factors that affect CLD. Even if the clinical signs recovered after ceasing wear, subclinical inflammation could persist. It was also suggested that changes in the immune environment of the ocular surface caused by long-term CL wear could have long-term effects, which possibly results in the inability to recover to prewear levels by discontinuing lens use.

## Data Availability Statement

The original contributions presented in the study are included in the article/Supplementary Material, further inquiries can be directed to the corresponding authors.

## Ethics Statement

The studies involving human participants were reviewed and approved by the Human Sciences Ethical Committee of the Eye Hospital at Wenzhou Medical University (approval number: KYK2018-23). The patients/participants provided their written informed consent to participate in this study.

## Author Contributions

YX, ZX, XS, QL, YW, JX, and YL collected, analyzed, and interpreted the data. YX, ZX, JQ, and LH were the major contributors for drafting the manuscript. All authors have read and approved the final manuscript.

## Funding

This work was supported by the Key Projects in Scientific Research Foundation of National Health Commission and Medical Science and Technology Program of Zhejiang Province (grant number: WKJ-ZJ-1930), the General Program in National Natural Science Foundation of China (grant number: 82070933), and the Science and Technology Program of Wenzhou (grant number: Y20160442).

## Conflict of Interest

The authors declare that the research was conducted in the absence of any commercial or financial relationships that could be construed as a potential conflict of interest.

## Publisher's Note

All claims expressed in this article are solely those of the authors and do not necessarily represent those of their affiliated organizations, or those of the publisher, the editors and the reviewers. Any product that may be evaluated in this article, or claim that may be made by its manufacturer, is not guaranteed or endorsed by the publisher.

## Supplementary Material

Supplementary Figure S1 The images of clinical signs and immune cells MGD, meibomian gland dysfunction; DC, dendritic cells. White arrows indicated DCs. Images of each clinical signs (each row) were captured from the same subject. All the DCs images were captured from the same subject at different time points.

Supplementary Table S1 The BAUSCH & LOMB^®^ SofLens 59^®^ physical/optical properties and lens parameters available. *: Oxygen Permeability value was determined by the polarographic method.

Supplementary Table S2 Demographics of the subjects (*n* = 26). SD, standard deviation; IQR, interquartile range; TBUT, tear film breakup time; OSDI, the Ocular Surface Disease Index; MGD, Meibomian gland dysfunction.

Supplementary Table S3 The changes of clinical signs, microvascular response, immune cells, and contact lens discomfort from baseline to 6 months post contact lens wear. Values were described as the mean ± SD (standard deviation). CL, contact lens; OSDI, the Ocular Surface Disease Index; TBUT, tear film breakup time; DC, dendritic cells; MGD, Meibomian gland dysfunction.

Supplementary Table S4 The effect of demographics, clinical signs, microvascular response, and immune cells on contact lens discomfort during contact lens wear (generalized estimating equation results). MGD, Meibomian gland dysfunction; TBUT, tear film breakup time; DC, dendritic cells; CI, confidence interval; OR, odds ratio. B and P: Generalized estimating equation (GEE), the Ocular Surface Disease Index (OSDI) scores as dependent variable, other indicators as independent variables, different time point as within-subject variables, working correlation matrix structure as Autoregressive.
