# Supplementary figures and images for: Dynamic Changes of Ocular Surface in First-Time Contact Lens Wearers and the Effective Factors of Contact Lens Discomfort
Source: Front Med (Lausanne). 2022 Mar 11;9:833962. doi: 10.3389/fmed.2022.833962 (PMC8962650; doi:10.3389/fmed.2022.833962)

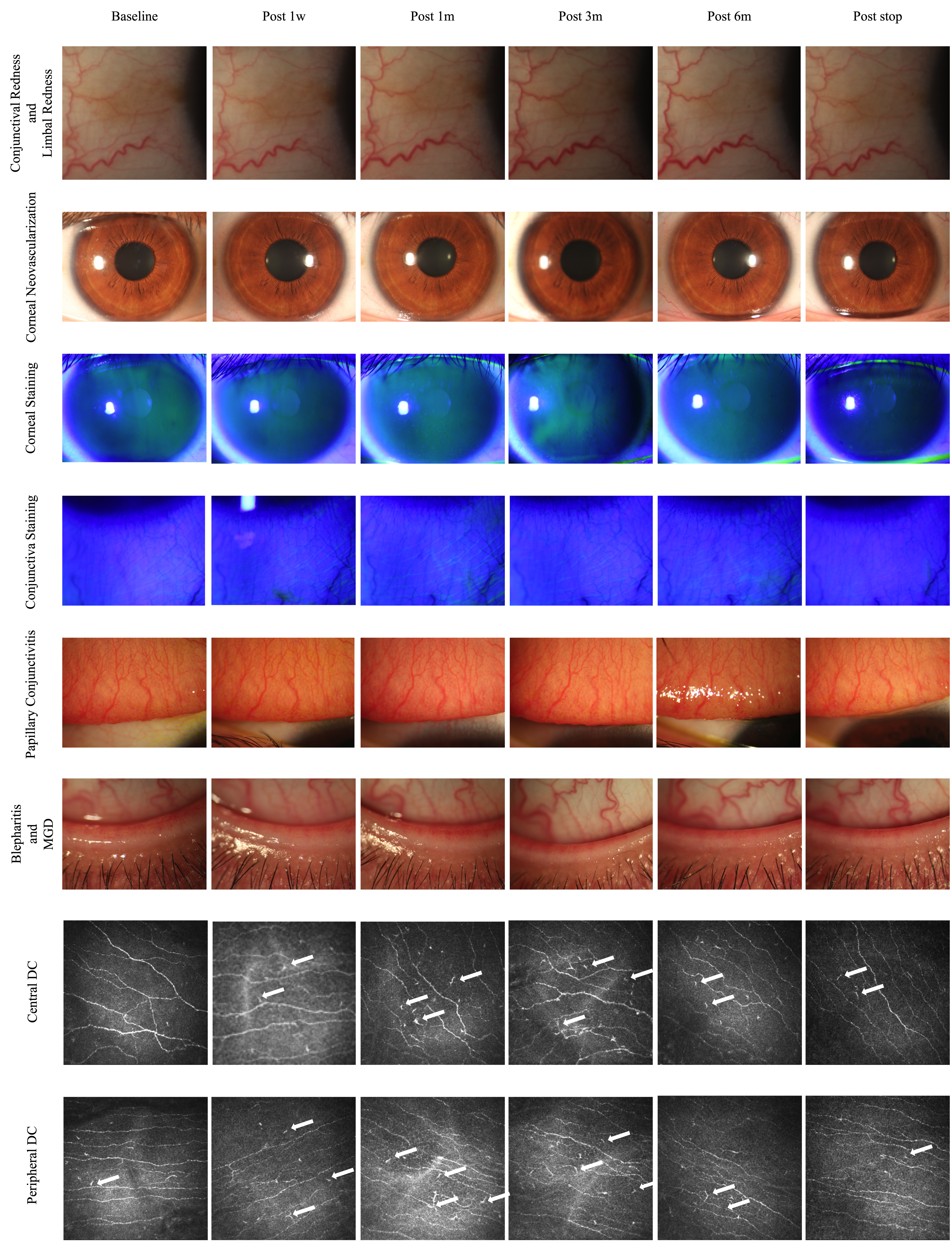

Supplement: Supplementary Figure S1 — The images of clinical signs and immune cells MGD, meibomian gland dysfunction; DC, dendritic cells. White arrows indicated DCs. Images of each clinical signs (each row) were captured from the same subject. All the DCs images were captured from the same subject at different time points. [file Image_1.TIFF]
