# Supplementary material for: Dynamic Changes of Ocular Surface in First-Time Contact Lens Wearers and the Effective Factors of Contact Lens Discomfort
Source: Front Med (Lausanne). 2022 Mar 11;9:833962. doi: 10.3389/fmed.2022.833962 (PMC8962650; doi:10.3389/fmed.2022.833962)
Supplement: Supplementary Table S1 — The BAUSCH & LOMB® SofLens 59® physical/optical properties and lens parameters available. *: Oxygen Permeability value was determined by the polarographic method. [file Table_1.docx]

**Supplementary Table 1**

The BAUSCH & LOMB^®^ SofLens 59^®^ physical / optical properties and lens parameters available.

| Variables | Value |
| --- | --- |
| Physical / optical properties |  |
| - Specific Gravity | 1.119 |
| - Refractive Index | 1.4036 |
| - Water Content | 59% |
| - Oxygen Permeability (Dk) | 22^*^ |
| Lens parameters available |  |
| - Diameter | 14.2mm |
| - Base Curve | 8.6mm |
| - Powers - Spherical | − 0.50D to − 6.00D in 0.25D steps  − 6.50D to − 9.00D in 0.50D steps |

^*^: Oxygen Permeability value was determined by the polarographic method.
