# Supplementary material for: Dynamic Changes of Ocular Surface in First-Time Contact Lens Wearers and the Effective Factors of Contact Lens Discomfort
Source: Front Med (Lausanne). 2022 Mar 11;9:833962. doi: 10.3389/fmed.2022.833962 (PMC8962650; doi:10.3389/fmed.2022.833962)
Supplement: Supplementary Table S2 — Demographics of the subjects (n = 26). SD, standard deviation; IQR, interquartile range; TBUT, tear film breakup time; OSDI, the Ocular Surface Disease Index; MGD, Meibomian gland dysfunction. [file Table_2.docx]

**Supplementary Table 2**

Demographics of the subjects (n = 26).

| Variables | Mean ± SD / Median (IQR) | Median | Min | Max |
| --- | --- | --- | --- | --- |
| Age, years | 23.69 ± 1.46 | 24 | 21 | 28 |
| Gender, Male/Female | 9/17 | - | - | - |
| Refractive error |  |  |  |  |
| - Spherical powers, D | -4.56 ± 2.22 | -4.63 | -0.25 | -8.50 |
| - Cylinder powers, D | -0.63 (0.75) | -0.63 | 0 | -1.50 |
| TBUT, s | 3.67 (4.25) | 3.67 | 1.00 | 15.67 |
| Schirmer's I test, mm | 18.10 (23.50) | 18.10 | 0.00 | 30.00 |
| OSDI, 0-100 points | 8.71 (6.90) | 8.71 | 0.00 | 31.25 |
| Efron Grading scales, 0-4 points |  |  |  |  |
| - Conjunctival redness | 1.36 ± 0.42 | 1.40 | 0.40 | 2.00 |
| - Limbal redness | 0.28 (0.38) | 0.28 | 0.00 | 1.60 |
| - Corneal neovascularization | 0.03 (0.07) | 0.03 | 0.00 | 0.33 |
| - Corneal staining | 0.23 (0.46) | 0.23 | 0.00 | 1.87 |
| - Conjunctival staining | 0.77 ± 0.40 | 0.70 | 0.10 | 1.50 |
| - Papillary conjunctivitis | 0.42 ± 0.25 | 0.40 | 0.10 | 0.93 |
| - Blepharitis | 0.47 ± 0.29 | 0.43 | 0.00 | 1.10 |
| - MGD | 0.70 (0.20) | 0.70 | 0.20 | 1.70 |
| Contact lens power - spherical, D | -4.41 ± 1.92 | -4.63 | -1.00 | -8.00 |

SD = standard deviation. IQR = interquartile range. TBUT = tear film breakup time. OSDI = the Ocular Surface Disease Index. MGD = Meibomian gland dysfunction.
