# Supplementary material for: Dynamic Changes of Ocular Surface in First-Time Contact Lens Wearers and the Effective Factors of Contact Lens Discomfort
Source: Front Med (Lausanne). 2022 Mar 11;9:833962. doi: 10.3389/fmed.2022.833962 (PMC8962650; doi:10.3389/fmed.2022.833962)
Supplement: Supplementary Table S3 — The changes of clinical signs, microvascular response, immune cells, and contact lens discomfort from baseline to 6 months post contact lens wear. Values were described as the mean ± SD (standard deviation). CL, contact lens; OSDI, the Ocular Surface Disease Index; TBUT, tear film breakup time; DC, dendritic cells; MGD, Meibomian gland dysfunction. [file Table_3.docx]

**Supplementary Table 3**

The changes of clinical signs, microvascular response, immune cells, and contact lens discomfort from baseline to 6 months post contact lens wear.

| Variables | Baseline  n = 26 | 1 week post CL wear  n = 26 | 1 month post CL wear  n = 26 | 3 months post CL wear  n = 21 | 6 months post CL wear  n = 21 | 6 months post stop wear  n = 13 |
| --- | --- | --- | --- | --- | --- | --- |
| **Clinical Signs** |  |  |  |  |  |  |
| Efron Grading scales, 0-4 points |  |  |  |  |  |  |
| - Conjunctival redness | 1.36 ± 042 | 1.43 ± 0.45 | 1.64 ± 0.45 | 1.51 ± 0.54 | 1.60 ± 0.58 | 1.45 ± 0.38 |
| - Limbal redness | 0.35 ± 0.39 | 0.73 ± 0.36 | 0.85 ± 0.39 | 0.80 ± 0.47 | 0.88 ± 0.51 | 0.34 ± 0.27 |
| - Corneal neovascularization | 0.06 ± 0.09 | 0.10 ± 0.13 | 0.14 ± 0.17 | 0.08 ± 0.06 | 0.08 ± 0.08 | 0.04 ± 0.04 |
| - Corneal staining | 0.42 ± 0.57 | 1.55 ± 0.89 | 1.79 ± 0.64 | 1.52 ± 0.88 | 1.34 ± 0.99 | 0.36 ± 0.45 |
| - Conjunctival staining | 0.77 ± 0.40 | 1.18 ± 0.54 | 1.19 ± 0.37 | 1.11 ± 0.12 | 0.90 ± 0.42 | 0.31 ± 0.21 |
| - Papillary conjunctivitis | 0.42 ± 0.25 | 0.64 ± 0.33 | 0.62 ± 0.29 | 0.73 ± 0.7 | 0.85 ± 0.36 | 0.51 ± 0.27 |
| - Blepharitis | 0.47 ± 0.29 | 0.51 ± 0.19 | 0.53 ± 0.17 | 0.51 ± 0.16 | 0.64 ± 0.24 | 0.24 ± 0.09 |
| - MGD | 0.74 ± 0.36 | 1.04 ± 0.33 | 0.91 ± 0.38 | 0.91 ± 0.39 | 1.09 ± 0.38 | 0.90 ± 0.38 |
| TBUT, s | 4.68 ± 3.22 | 3.87 ± 2.49 | 3.23 ± 1.46 | 3.73 ± 2.13 | 3.44 ± 1.60 | 4.90 ± 3.24 |
| Schirmer's I test, mm | 16.68 ± 11.47 | 12.97 ± 9.38 | 14.17 ± 9.96 | 13.44 ± 10.50 | 17.96 ± 10.76 | 14.95 ± 9.64 |
| **Conjunctival Microvascular Response** |  |  |  |  |  |  |
| Axial blood flow velocity (Va), mm/s | 0.51 ± 0.13 | 0.54 ± 0.13 | 0.59 ± 0.16 | 0.55 ± 0.14 | 0.55 ± 0.11 | 0.57 ± 0.15 |
| Vessel density (Dbox) | 1.66 ± 0.05 | 1.67 ± 0.08 | 1.66 ± 0.05 | 1.66 ± 0.07 | 1.63 ± 0.06 | 1.62 ± 0.06 |
| **Immune Cells** |  |  |  |  |  |  |
| Central cornea |  |  |  |  |  |  |
| - DC density, cells/mm^2^ | 22.28 ± 18.96 | 32.33 ± 25.32 | 56.92 ± 47.26 | 52.71 ± 38.47 | 58.33 ± 45.19 | 62.82 ± 46.20 |
| - DC area, μm^2^ | 52.75 ± 17.27 | 60.35 ± 16.73 | 66.49 ± 13.64 | 68.11 ± 16.03 | 62.45 ± 8.59 | 64.57 ± 11.16 |
| - number of dendrites per DC, No. | 2.25 ± 0.22 | 2.45 ± 0.27 | 2.56 ± 0.25 | 2.54 ± 0.28 | 2.58 ± 0.22 | 2.58 ± 0.47 |
| Peripheral cornea |  |  |  |  |  |  |
| - DC density, cells/mm^2^ | 33.49 ± 38.58 | 59.05 ± 41.68 | 84.70 ± 49.26 | 65.18 ± 30.76 | 59.13 ± 48.53 | 57.21 ± 39.70 |
| - DC area, μm^2^ | 66.20 ± 19.27 | 83.70 ± 24.13 | 101.11 ± 26.25 | 88.04 ± 21.81 | 67.81 ± 20.57 | 71.60 ± 14.13 |
| - number of dendrites per DC, No. | 2.52 ± 0.27 | 2.71 ± 0.34 | 2.98 ± 0.44 | 2.72 ± 0.31 | 2.51 ± 0.27 | 2.65 ± 0.34 |
| **Subjective Comfort** |  |  |  |  |  |  |
| OSDI, 0-100 points | 9.68 ± 7.66 | 24.25 ± 10.28 | 24.98 ± 12.45 | 24.02 ± 14.80 | 24.41 ± 12.01 | 16.11 ± 7.31 |

Values were described as the mean ± SD (standard deviation). CL = contact lens. OSDI = the Ocular Surface Disease Index. TBUT = tear film breakup time. DC = dendritic cells. MGD = Meibomian gland dysfunction.
