# Supplementary material for: Dynamic Changes of Ocular Surface in First-Time Contact Lens Wearers and the Effective Factors of Contact Lens Discomfort
Source: Front Med (Lausanne). 2022 Mar 11;9:833962. doi: 10.3389/fmed.2022.833962 (PMC8962650; doi:10.3389/fmed.2022.833962)
Supplement: Supplementary Table S4 — The effect of demographics, clinical signs, microvascular response, and immune cells on contact lens discomfort during contact lens wear (generalized estimating equation results). MGD, Meibomian gland dysfunction; TBUT, tear film breakup time; DC, dendritic cells; CI, confidence interval; OR, odds ratio. B and P: Generalized estimating equation (GEE), the Ocular Surface Disease Index (OSDI) scores as dependent variable, other indicators as independent variables, different time point as within-subject variables, working correlation matrix structure as Autoregressive. [file Table_4.docx]

**Supplementary Table 4**

The effect of demographics, clinical signs, microvascular response, and immune cells on contact lens discomfort during contact lens wear (generalized estimating equation results).

| Variables | *B* | Standard error | *P* | 95% CI | OR | OR 95% CI |
| --- | --- | --- | --- | --- | --- | --- |
| **Demographics** |  |  |  |  |  |  |
| Age | 0.102 | 0.104 | 0.328 | -0.102 to 0.305 | 1.107 | 0.903 to 1.357 |
| Contact lens power - spherical | 0.324 | 0.145 | **0.026** | 0.039 to 0.608 | 1.382 | 1.040 to 1.837 |
| **Clinical Signs** |  |  |  |  |  |  |
| Efron Grading scales |  |  |  |  |  |  |
| - Conjunctival redness | -0.061 | 0.073 | 0.402 | -0.203 to 0.081 | 0.941 | 0.816 to 1.085 |
| - Limbal redness | 0.100 | 0.079 | 0.208 | -0.055 to 0.255 | 1.105 | 0.946 to 1.290 |
| - Corneal neovascularization | 0.026 | 0.103 | 0.802 | -0.175 to 0.227 | 1.026 | 0.839 to 1.255 |
| - Corneal staining | 0.238 | 0.077 | **0.002** | 0.087 to 0.389 | 1.268 | 1.090 to 1.476 |
| - Conjunctival staining | 0.013 | 0.079 | 0.874 | -0.143 to 0.168 | 1.013 | 0.867 to 1.183 |
| - Papillary conjunctivitis | 0.245 | 0.062 | **<0.001** | 0.124 to 0.367 | 1.278 | 1.132 to 1.443 |
| - Blepharitis | 0.021 | 0.054 | 0.701 | -0.085 to 0.127 | 1.021 | 0.918 to 1.135 |
| - MGD | 0.059 | 0.090 | 0.510 | -0.117 to 0.235 | 1.061 | 0.890 to 1.265 |
| TBUT | 0.087 | 0.062 | 0.161 | -0.035 to 0.209 | 1.091 | 0.966 to 1.232 |
| Schirmer's I test | 0.138 | 0.084 | 0.101 | -0.027 to 0.303 | 1.148 | 0.973 to 1.354 |
| **Conjunctival Microvascular Response** |  |  |  |  |  |  |
| Axial blood flow velocity (Va) | 0.353 | 0.076 | **<0.001** | 0.204 to 0.502 | 1.423 | 1.226 to 1.652 |
| Vessel density (Dbox) | 0.078 | 0.068 | 0.252 | -0.056 to 0.212 | 1.081 | 0.946 to 1.236 |
| **Immune Cells** |  |  |  |  |  |  |
| Central cornea |  |  |  |  |  |  |
| - DC density, cells/mm^2^ | 0.106 | 0.066 | 0.108 | -0.023 to 0.236 | 1.112 | 0.977 to 1.266 |
| - DC area, μm^2^ | 0.006 | 0.068 | 0.934 | -0.129 to 0.140 | 1.006 | 0.879 to 1.150 |
| - number of dendrites per DC, No. | -0.043 | 0.079 | 0.589 | -0.198 to 0.112 | 0.958 | 0.821 to 1.119 |
| Peripheral cornea |  |  |  |  |  |  |
| - DC density, cells/mm^2^ | -0.181 | 0.103 | 0.079 | -0.384 to 0.021 | 0.834 | 0.681 to 1.021 |
| - DC area, μm^2^ | 0.048 | 0.067 | 0.479 | -0.084 to 0.180 | 1.049 | 0.919 to 1.197 |
| - number of dendrites per DC, No. | -0.007 | 0.058 | 0.902 | -0.122 to 0.107 | 0.993 | 0.885 to 1.113 |

MGD = Meibomian gland dysfunction. TBUT = tear film breakup time. DC = dendritic cells. CI = confidence interval. OR = odds ratio. *B* and *P*: Generalized estimating equation (GEE), the Ocular Surface Disease Index (OSDI) scores as dependent variable, other indicators as independent variables, different time point as within-subject variables, working correlation matrix structure as “Autoregressive”.
